# Impedance-based Real-time Monitoring of Neural Stem Cell Differentiation

**DOI:** 10.2478/joeb-2021-0006

**Published:** 2021-11-20

**Authors:** F. J. Shah, C. Caviglia, K. Zór, M. Carminati, G. Ferrari, M. Sampietro, A. Martínez-Serrano, J. K. Emnéus, A. R. Heiskanen

**Affiliations:** 1 Department of Micro- and Nanotechnology, Technical University of Denmark, Produktionstorvet, Building 423, 2800 Kongens Lyngby, Denmark; 2 Particle Analytical ApS, Agern Allé 3, 2970 Hørsholm, Denmark; 3 Radiometer Medical ApS, Åkandevej 21, 2700 Brønshøj, Denmark; 4 Center for Intelligent Drug Delivery and Sensing Using Microcontainers and Nanomechanics, Department of Health Technology, Technical University of Denmark, Ørsteds Plads, Building 345C, 2800 Kongens Lyngby, Denmark; 5 Dipartimento di Elettronica, Informazione e Bioingegneria - DEIB, Politecnico di Milano, P.za L. da Vinci 32, 20133 Milano, Italy; 6 Department of Molecular Neuropathology, Center of Molecular Biology Severo Ochoa, Universidad Autónoma de Madrid, Calle Nicolás Cabrera 1, Cantoblanco, 28049 Madrid, Spain; 7 Present affiliation: Department of Biotechnology and Biomedicine, Technical University of Denmark, Produktionstorvet, Building 423, 2800 Kongens Lyngby, Denmark

**Keywords:** Neural stem cell, Dopaminergic neuron, Stem cell differentiation, ECIS, IDE, Impedance, Equivalent circuit

## Abstract

We present here the first impedance-based characterization of the differentiation process of two human mesencephalic fetal neural stem lines. The two dopaminergic neural stem cell lines used in this study, Lund human mesencephalic (LUHMES) and human ventral mesencephalic (hVM1 Bcl-X_L_), have been developed for the study of Parkinsonian pathogenesis and its treatment using cell replacement therapy. We show that if only relying on impedance magnitude analysis, which is by far the most usual approach in, e.g., cytotoxicity evaluation and drug screening applications, one may not be able to distinguish whether the neural stem cells in a population are proliferating or differentiating. However, the presented results highlight that equivalent circuit analysis can provide detailed information on cellular behavior, e.g. simultaneous changes in cell morphology, cell-cell contacts, and cell adhesion during formation of neural projections, which are the fundamental behavioral differences between proliferating and differentiating neural stem cells. Moreover, our work also demonstrates the sensitivity of impedance-based monitoring with capability to provide information on changes in cellular behavior in relation to proliferation and differentiation. For both of the studied cell lines, in already two days (one day after induction of differentiation) equivalent circuit analysis was able to show distinction between proliferation and differentiation conditions, which is significantly earlier than by microscopic imaging. This study demonstrates the potential of impedance-based monitoring as a technique of choice in the study of stem cell behavior, laying the foundation for screening assays to characterize stem cell lines and testing the efficacy epigenetic control.

## Introduction

Parkinson’s disease (PD) is a progressive neurodegenerative disorder mainly affecting the motor functions due to dysfunctional or dying dopamine (DA) producing neurons in substantia nigra pars compacta [1]. Currently, the applied therapeutic approaches include, e.g., L-3,4-dihydroxyphenylalanine (L-DOPA) medication, administration of a DA receptor agonist, and electrical deep brain stimulation in the subthalamic nucleus. All of them are symptomatic treatments with several limitations and implicated by side effects causing motor response oscillations as well as L-DOPA induced dyskinesia [2]. To restore DA production in PD patients, one of the suggested therapeutic approaches is cell replacement therapy (CRT), i.e. transplantation of cells that acquire dopaminergic properties in the brain [1,2]. Stem cell lines of different origins have been exploited and investigated as relevant cellular sources for CRT in PD. The stem cells can be further categorized into embryonic stem cells, neural stem cells (NSCs), induced neural stem cells, mesenchymal stem cells, and induced pluripotent stem cells, each type having both advantages and disadvantages. NSCs from fetal mesencephalon are considered to be one of the potential sources of transplantable cells [1,2]. However, NSCs must be differentiated into dopaminergic neurons prior to transplantation in order to avoid inefficient transplantation as well as possible tumorigenicity [2]. In addition, the purity and yield of cells having dopaminergic phenotype are critical for successful stem cell-based CRT. For these reasons, *in vitro* characterization of the differentiation process of stem cells is highly important for future development of therapies.

Several biochemical assays are available to monitor and characterize stem cell differentiation *in vitro*, e.g., reverse transcription polymerase chain reaction, immunocyto-chemistry, Western/Northern/Southern blotting, and flow cytometric analysis for particular markers [3, 4, 5, 6, 7]. These methods have contributed to improved understanding of stem cells at the molecular level. However, none of the above mentioned assays are able to characterize cells without fixation, staining, lysing, or fluorescent labelling, only providing possibility to perform characterization on separate cell populations as an end-point after a varying period of differentiation [8]. To overcome these limitations, label-free non-invasive real-time monitoring approaches, which allow continuous assessment of each cell population with time without affecting the biological and therapeutic functionality of the cells, are needed [9].

Having the diverse applications of stem cells on the horizon, the need for non-invasive real-time characterization may comprise, e.g., high-content and high-throughput screening for quality and safety assessment in large-scale production of stem cells for CRT and drug development, as well as for disease modeling, pharmacological studies, and toxicity assessment [10]. Real-time bright-field and epifluorescence imaging as well as molecular beacon-based monitoring of mRNA appearance can provide valuable information about changes in cellular morphology and gene expression, respectively [11, 12, 13]. However, especially electrical impedance measurements, pioneered by Giaever and Keese [14], have become well-established as a label-free real-time monitoring technique that has found many applications in monitoring of complex cellular behavior, such as cell motility [15], adhesion and spreading [16], proliferation and cytotoxicity [17,18] as well as receptor activation [19,20]. Additionally, due to the increased interest for studying stem cell differentiation [4], impedance monitoring has proved to be a useful and powerful tool also in real-time monitoring of this cellular processes, revealing mechanisms, the study of which otherwise requires laborious end-point assays [8, 9, 10,21,22].

Two human mesencephalic fetal neural stem lines, LUHMES [23,24] and hVM1 Bcl-X_L_ [25,26], developed for applications concerning the pathogenesis and CRT in PD, have been thoroughly characterized at different stages of differentiation in terms of their gene expression and ability to acquire dopaminergic phenotype [12,13,23, 24, 25, 26, 27, 28, 29]. In this study, we demonstrate how impedance-based multi-parameter analysis can serve as a tool for distinguishing proliferating cell populations from ones undergoing differentiation. We also present a comparison between the widely used data analysis approach to monitor changes in normalized impedance magnitude with time and equivalent circuit analysis. The impedance-based findings are correlated with fluorescence microscopic visualization of morphological changes during the progress of proliferation and differentiation.

## Materials and methods

### Measurement setup and preparation of experiments

**Figure 1:**
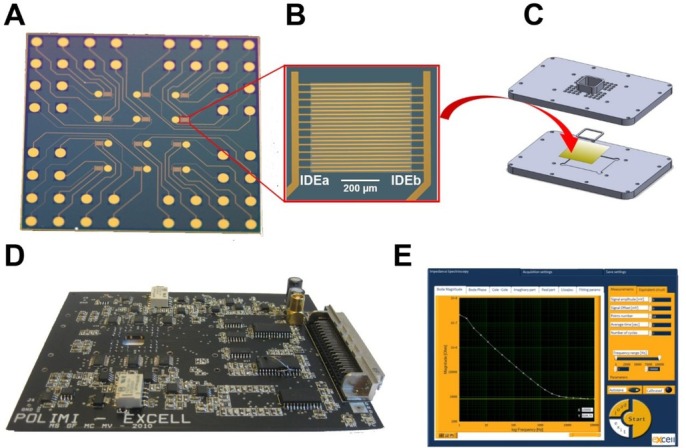
Impedance measurement setup: **A**) Microelectrode array chip with 12 IDEs; **B**) zoom-in view of one IDE (the ca. 500 μm x 500 μm opening in the silicon nitride passivation layer appears as a lighter region in the center) ; **C**) chip holder (the lower plate accommodates a MEA chip and the upper plate provides the 600 μl cell culture chamber and an array of holes for electrical connections using spring-loaded pins; fluid tight sealing on the MEA chip is achieved by using a laser cut silicon rubber gasket); **D**) Printed circuit board (PCB) of the custom-made 12-channel bipotentiostat (the PCB has an opening to the cell culture vial of the chip holder to allow liquid handling and microscopic visualization; **E**) user interface of the data acquisition software showing recorded impedance magnitude vs. log frequency.

All experiments were performed using a previously reported impedance measurement setup (Figure 1) comprising a micromilled poly(methyl methacrylate) cell culture unit (600 μL well for cell culturing) [17], having a microelectrode array (MEA) chip with 12 interdigitated electrodes (IDEs – 12 digits: length 500 μm; width and gap 10 μm), fabricated in a previously published UV lithographic process including e-beam evaporation of metals (150 nm of Au on a 10 nm Ti adhesion layer; 500 nm silicon nitride passivation layer to define active electrode areas and contact pads) [30], a miniaturized custom-made 12-channel bipotentiostat and data acquisition software [31].

Prior to cell seeding in the cell culture device for impedance-based assays, each MEA chip was cleaned using the previously described two-step method, including a chemical (10 min in a mixture of 25% H_2_O_2_ and 50 mM KOH) and electrochemical (potential sweep in 50 mM KOH between −200 mV and −1200 mV) step [32]. Sterilization of the culture well was done by a 20-minute treatment with 500 mM NaOH followed by thorough rinsing with PBS [17]. By using the abovementioned cleaning procedure, each microelectrode chip could be reused in three experiments.

### Cell lines and culture conditions

Lund human mesencephalic (LUHMES) [23,24] and human ventral mesencephalic (hVM1 Bcl-X_L_) [25,26] neural stem cell lines were generated using human ventral mesencephalic tissue from 8 and 10 weeks old fetuses, respectively. Both cell lines were immortalized using the v-*myc* oncogene [23,26]. LUHMES cells were purchased from ATCC (CRL-2927). hVM1 Bcl-X_L_ cells, developed in the laboratory of A.M.-S., overexpress Bcl-X_L_, which protects the cells against apoptosis, and enhances the generation of β-III-tubulin and tyrosine hydroxylase positive cells, i.e. DA neurons, during differentiation [26]. hVM1 Bcl-X_L_ cells proliferate in the presence of epidermal (EGF) and basic fibroblast (bFGF) growth factor. When the mitogens are withdrawn the expression of v-*myc* is reduced and the initiation of differentiation process into neurons, oligo-dendrocytes and astrocytes is induced [25,26].

Both cell lines were cultured in Nunc flasks that were pre-coated with Geltrex® (ThermoFisher, A1413301), diluted 1:100 in sterile PBS, at 37 °C for 1 h. After the coating, Geltrex® was removed and cells were seeded and cultured at 37 °C in a humidified incubator (5% CO_2_ in air) using Dulbecco’s modified Eagle’s medium/F12 with GlutaMax (DMEM/F12/GlutaMax) containing 0.5 % AlbuMax-I, 1 % Penicillin/Streptomycin (P/S), 1 % N2 supplement (all purchased from Thermofisher Scientific), as well as for LUHMES: 40 ng/ml human recombinant basic fibroblast growth factor (hrbFGF, R&D systems, USA), and for hVM1 Bcl-X_L_: 20 ng/ml hrbFGF; 20 ng/ml human recombinant epidermal growth factor (hrEGF, R&D systems, USA).

Different cell densities (30,000 cells/cm^2^, 60,000 cells/cm^2^ and 120,000 cells/cm^2^) were seeded onto a Geltrex^®^ pre-coated electrode array chip. For differentiation experiments, 24 hours after cell seeding the culture medium was replaced by differentiation medium prepared in DMEM/F12/GlutaMax containing 0.5 % AlbuMax-I, 1 % P/S, 1 % N2 supplement, 1 mM cyclic adenosine monophosphate (cAMP, Sigma-Aldrich, USA), as well as for LUHMES: 1 μg/ml tetracycline (Sigma-Aldrich, USA), 40 ng/ml human recombinant glial cell line derived neurotrophic factor (hrGDNF, R&D systems, USA) and for hVM1 Bcl-X_L_: 2 ng/ml hrGDNF. During differentiation, half of the medium was changed every second day.

### Microscopic imaging

End-point staining was performed to evaluate viability of the cells. A stock solution of 1 mg/ml Calcein AM (Sigma Aldrich, USA) was prepared in DMSO (Sigma Aldrich, USA) and diluted to 2 μg/ml in PBS prior to use. Staining was performed for 10 min at 37 °C. The cells were further incubated in fresh medium for 10 min to allow de-esterification of AM esters. Fluorescence microscopy images were acquired using an LSM 700 confocal laser scanning microscope (Carl Zeiss AG, Göttingen, Germany) and the ZEN lite software (Carl Zeiss AG, Göttingen, Germany). The employed laser light source provided the 488 nm excitation wavelength. Emission was monitored at 516 nm.

### Impedance measurements

Impedance spectra (30 data points in the frequency range from 100 Hz to 100 kHz with the averaging time of 2 s) were recorded continuously at a time interval of 4 h on each of the 12 IDEs of an electrode array chip over the entire experimental period. The applied sinusoidal potential was set to 200 μV. The impedance measurements were performed using the bipolar sensing configuration facilitated by IDEs to achieve higher sensitivity as previously demonstrated [33].

### Data analysis and presentation

**Figure S1:**
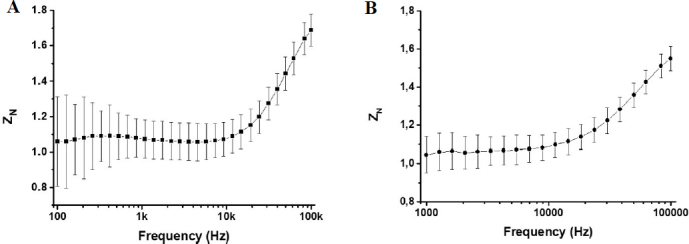
Normalized impedance vs. frequency for **A**) LUHMES (60,000 cells/cm^2^) and **B**) hVM1 Bcl-X_L_ (120,000 cells/cm^2^). The spectra were acquired 48 h after cell seeding. (Error bars: standard deviation, n = 3)

Changes in the recorded impedance were presented using the dimensionless parameter, Cell Index (CI), which represents the maximum value of the background subtracted normalized impedance based on Equation (1) [34],


(1) $$Cell\;Index\;(t)=max_{i=1,\ldots ,N}\frac{\left|Z\left(t,f_{i}\right)\right|-\left|Z_{0}\left(f_{i}\right)\right|}{\left|Z_{0}\left(f_{i}\right)\right|}$$

where |*Z(t,fi)*| is the impedance magnitude at a given frequency and time point and |*Z0(fi)*| is the impedance magnitude at the same frequency recorded in the absence of cells at the beginning of the experiment. For each time point, the CI was calculated by analyzing the entire spectrum (N = 30). Matlab (R2013a) (MathWorks®, Natick, MA, USA) was used to create specific algorithms for data processing and analysis. The CI was calculated at 100 kHz, which was found to be the frequency corresponding to the most sensitive region of the spectra (Supplementary Material Figure S-1). At this frequency, the impedance magnitude is influenced by the cell membrane resistance and extracellular resistance as well as membrane capacitance, which still form the primary contribution even at frequencies up to 1 MHz [17].

Equivalent circuit-based data analysis was done using EchemAnalyst software (V. 6.10) from Gamry Instruments (Warminster, USA) by fitting the data to the appropriate equivalent circuit models using nonlinear least squares regression. To secure sufficient cell density on the analyzed electrodes, equivalent circuit-based data analysis was performed only for impedance spectra acquired on electrodes that showed at least 10 % increase in CI during the first 24 h.

For each experiment, the acquired impedance data were analyzed and averaged. Each experiment was repeated at least twice. Data are presented as average ± standard error of mean (s.e.m.) unless otherwise stated. Comparisons between means were performed using t-test (Prism 9, GraphPad Software, San Diego, CA, USA). Significant differences between means were indicated by asterisk: p < 0.05 (*); p < 0.01 (**); p < 0.001 (***), p < 0.0001 (****).

### Ethical approval

The conducted research is not related to either human or animal use.

## Results and discussion

In cell-based applications, impedance measurements were originally demonstrated using a configuration with small sensor electrodes and a large distant counter electrode [14].

This format is still widely used in many studies based on either custom-made or commercial setups. Ehret *et al*. were among the first ones to present cell-based impedance measurements using interdigitated electrodes (IDE) that have a pair of adjacent (equidistant) electrodes with equal dimensions [35]. Later, IDEs have become a standard also in commercial impedance measurement setups. In the study of Ehret *et al*., the rational of using IDEs was to minimize the impedance contribution of the bulk solution. Further applications based on IDEs have demonstrated higher sensitivity in cell-based impedance measurements [33,36]. It is worth to note that when performing impedance measurements using the combination of a large counter electrode and a small sensor electrode, the interface impedance of the large electrode is negligible. In the case of IDEs, on the other hand, the interface impedance of both sides, if covered by cells, have equal contribution to the measured impedance.

The most usual approach to present changes in impedance is to use either normalized impedance magnitude [15] or Cell Index (CI) [34], which is background subtracted normalized impedance magnitude based on the frequency that provides the highest sensitivity. In most studies related to, for instance, toxicological evaluations and also drug testing, this approach provides sufficient information on the time-course of cellular responses.

Impedance data, comprising the magnitude and phase angle, can be presented, aside from the normalized magnitude, using the real and/or imaginary component of impedance evaluated in the complex impedance plane (Nyquist format), complex admittance plane (inverse of impedance), or complex capacitance (angular frequency normalized admittance) plane (also denoted as complex dielectric permittivity plane) [37]. By presenting changes in any of these parameters with time can provide additional information of cellular interaction with the electrode surface and, hence, how the cellular functions and integrity are affected by different chemicals or biochemical processes. This approach was applied by, for instance, Bagnaninchi and Drummond in their study on differentiation of adipose-derived stem cells [9]. If recording complete impedance spectra at different time points, the possible third approach for analyzing impedance data in cell-based applications is to use an equivalent circuit model that describes the spectral behavior and is relevant for the cellular system. Although not as usual as presentation of normalized impedance as a function or time, this approach has been used in many investigations to obtain more details regarding cellular behavior.

In the study presented here, our focus was to obtain information that would allow differentiation between proliferating and differentiating neural stem cells. We evaluated the potentials of both normalized impedance presentation and equivalent circuit analysis, both of which are presented below.

### Normalized impedance magnitude analysis for discrimination of proliferating and differentiating neural stem cells

#### The behavior of LUHMES cells

The LUHMES cell line is one of the most commonly used immortalized cell lines as a model for Parkinson’s disease [38]. The cells express v-*myc* under control of tetracycline [23]. In the absence of tetracycline, v-*myc* expression allows the cells to proliferate into a homogenous population, while addition of tetracycline terminates the expression of v-*myc*, inducing neuronal differentiation into postmitotic neurons expressing specific markers of dopaminergic neurons and develop long neural projections [24,39]. Functionally, the differentiated cells display electrophysiological properties of dopaminergic neurons and are able to release DA [40].

**Figure 2:**
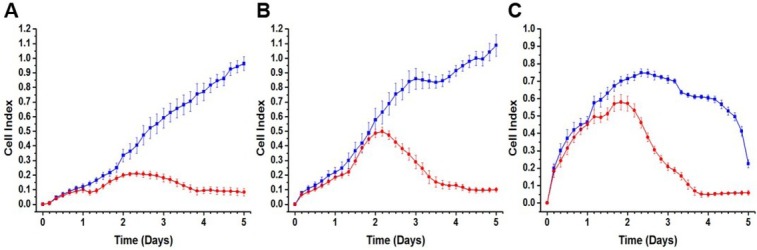
Cell Index vs. time for LUHMES cells: Initial cell density (cells/cm^2^) **A**) 30,000; **B**) 60,000; **C**) 120,000. Proliferating (blue) and differentiating (red) cells. Time in days after cell seeding. (Error bars: s.e.m., n = 6).

Initially, the correlation between cell density and CI was investigated by continuous impedance monitoring of LUHMES cells for 5 days at three different cell densities (30,000 cells/cm^2^, 60,000 cells/cm^2^, and 120,000 cells/cm^2^) under proliferation and differentiation conditions. The cells were seeded on the electrodes at time zero. Figure 2 shows growth and differentiation profiles of LUHMES cells at all three cell densities. For proliferating cells at 30,000 cells/cm^2^ and 60,000 cells/cm^2^ (Figure 2A and B), a steady increase in CI was observed over the 5-day period. For the highest cell density, 120,000 cells/cm^2^ (Figure 2C), the CI increased and levelled off upon reaching confluent cell layer followed by a drop, indicating oversaturation of the culture well and spontaneous detachment of the cells. The corresponding behavior has been previously reported for cancer cells and explained as immediate complete cell coverage on the electrode surface causing weak adhesion of the cells [17].

**Figure 3:**
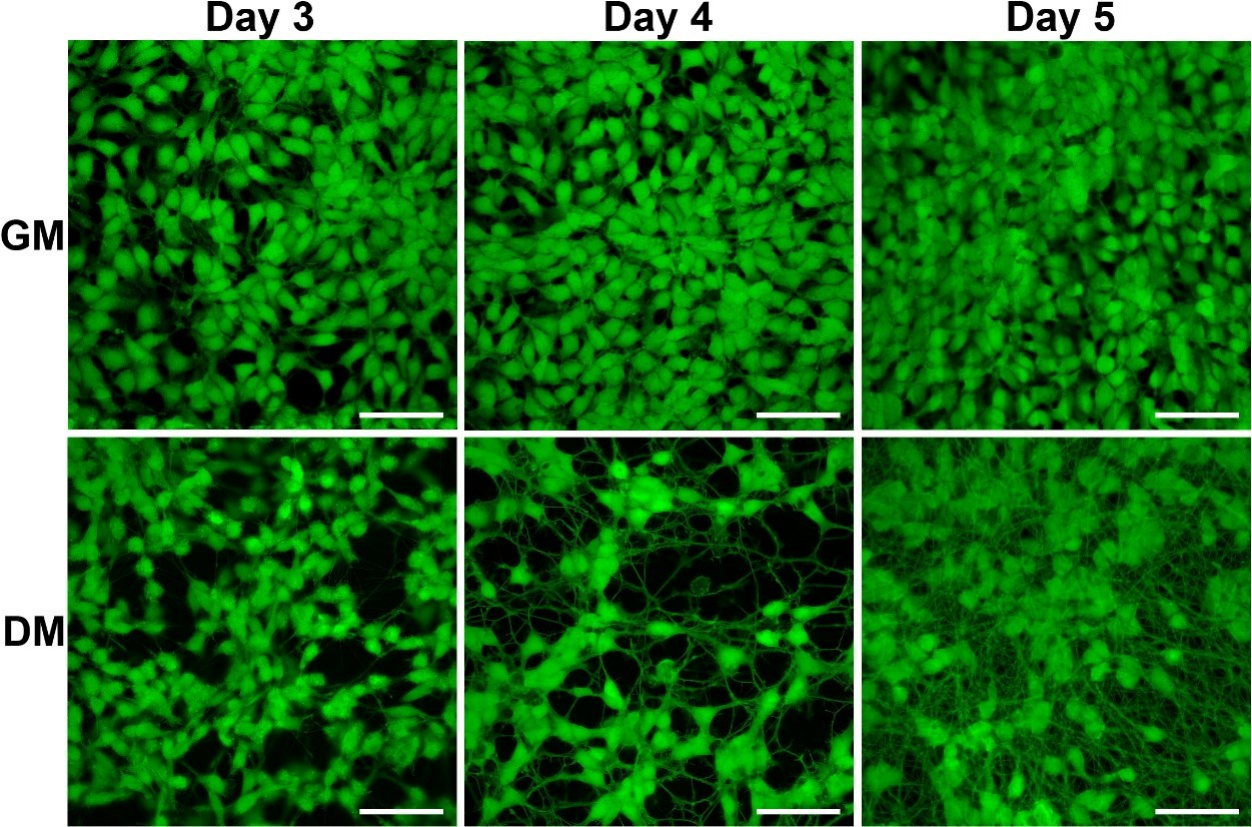
Fluorescence microscopy images of live stained (Calcein AM) LUHMES cells in growth medium (GM) and differentiation medium (DM). Initial cell density 60,000 cells/cm^2^. Time in days after cell seeding. (Scale bars: 50 μm).

Differentiation of LUHMES cells was initiated at day 1. The CI for the differentiating cells followed initially the same pattern as for the proliferating cells. On day 2, the CI for differentiating cells started to decrease and continued to decrease over time until it leveled off around day 4, reaching approximately the same level for all the cell densities. Based on microscopic imaging, this decrease in CI could be attributed to the morphological changes of the cells during differentiation (Figure 3). The choice of the optimal cell density for subsequent experiments and equivalent circuit-based data analysis was determined based on the observed behavior of the CI for both proliferating and differentiating cells at the three cell densities. At the density of 60,000 cells/cm^2^, proliferation could be monitored throughout the entire 5-day period without any decrease whereas differentiation caused a clear change in the CI at a distinct time point. Hence, this was considered as the most optimal density for LUHMES cells.

Additionally, we acquired images of live stained (calcein AM) LUHMES cells during days 3-5 (Figure 3) to confirm that impedance monitoring did not affect cell viability. On day 3, no obvious changes in the morphology of the calcein stained cells could be observed. However, on day 4, the differentiating cells showed decrease in size and increased formation of neural projections. On day 5, all the differentiating cells showed manifestation of a similar morphology with several long processes protruding from the cell soma, whereas the proliferating cells started to become round and overgrow each other.

Based on the CI profile, an early discrimination between growing and differentiating cells was possible (Figure 2). As early as day 2, i.e. 1 day after induction of neural differentiation, the behavior of differentiating cells started to divert from that of the proliferating cells. On day 3, the CI value for the proliferating cells had increased to 0.9, whereas the corresponding value for the differentiating cells had decreased to 0.3. The CI profiles for proliferating and differentiating LUHMES cells, shown in Figure 2A and B, present cellular behavior that is similar to what has been shown previously by Bagnaninchi and Drummond for adipose-derived stem cells differentiating to either osteoblasts or adipocytes [9]. For the cells that underwent osteogenesis, impedance at 64 kHz continued to increase steadily after induction of differentiation.

On the other hand, adipogenesis led to decrease in the recorded impedance soon after induction of adipogenesis and later on leveled off at values slightly above the initial impedance at the time of cell seeding. In that study, phase-contrast imaging indicated that osteogenesis led to decreased cell size and tightened cell-cell junctions, whereas adipogenesis caused the opposite effect. Based on fluorescence microscopy in our study, images shown in Figure 3, the decrease in CI could be attributed to the decrease in cell size and formation of neural projections, both of which contribute to an increase in the free electrode area. However, on day 3, the changes in the cellular morphology that can be seen in microscope images are still very weakly manifested. Hence, the overall conclusion is that impedance monitoring was able to reveal changes in the behavior of LUHMES cells clearly earlier than microscopy. Comparison between our study and differentiation of adipose-derived stem cells [9] shows clearly that although the normalized impedance or the recorded changes in impedance magnitude may behave similarly for different cell lines, the underlying reasons in terms of cell morphology and cell-cell contacts may differ.

#### The behavior of hVM1 Bcl-X_L_ cells

The hVM1 Bcl-X_L_ cell line has been differentiated into functional A9 type substantia nigra dopaminergic neurons that exert morphological and functional properties similar to the ventral mesencephalon primary neurons [41]. This cell line has also been transplanted in parkinsonian animals showing amelioration of motor functions in short- and longterm studies [42,43].

**Figure S2:**
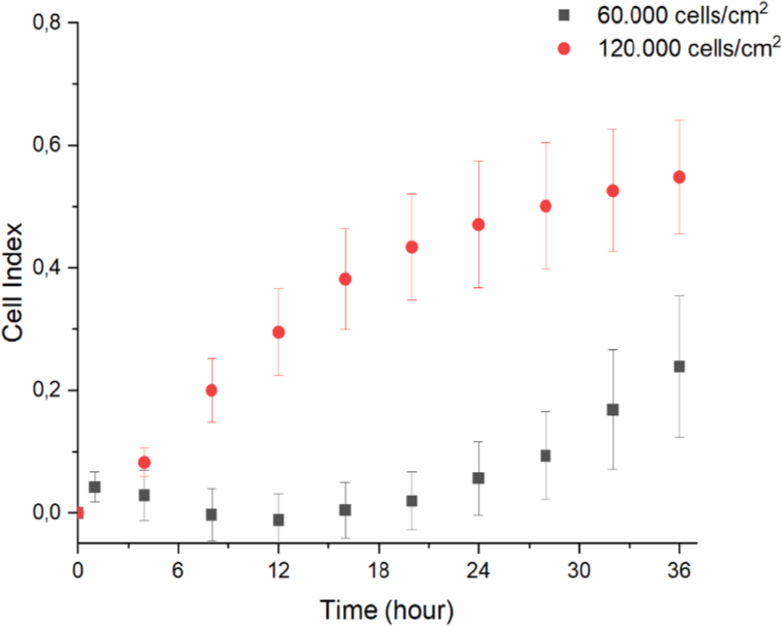
Cell Index vs. time for proliferating hVM1 Bcl-X_L_ cells. Initial cell seeding density 60,000 cells/cm^2^ and 120,000 cells/cm^2^. (Error bars: standard deviation, n = 3)

Prior to performing impedance monitoring of proliferating and differentiating hVM1 Bcl-X_L_ cells, the optimal cell density was determined by performing preliminary 36 h long impedance monitoring of proliferating cells seeded at two different densities, 60,000 cells/cm^2^ and 120,000 cells/cm^2^. As shown in the Supplementary Material Figure S-2, the CI for 120,000 cells/cm^2^ showed a clear increase immediately after cell seeding and reached the value of over 0.5 after 36 h, whereas the CI for 60,000 cells/cm^2^ indicated a long lag phase and weak increase after that. Hence, 120,000 cells/cm^2^ was chosen for the subsequent experiments.

**Figure 4:**
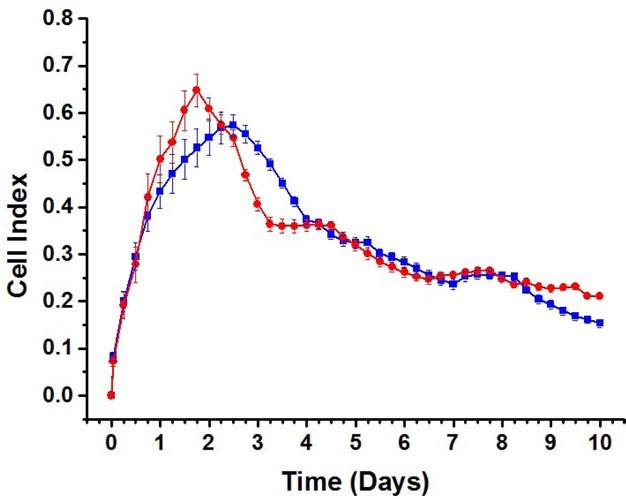
Cell Index vs. time for hVM1 Bcl-X_L_ cells: Initial cell density 120,000 cells/cm^2^. Proliferating (blue) and differentiating (red) cells. Time in days after cell seeding. (Error bars: s.e.m., n = 6).

Impedance monitoring of proliferating and differentiating hVM1 Bcl-X_L_ cells was performed for 10 days (Figure 4). The cells were seeded at day 0 and at day 1 the differentiation was induced. For both conditions, the CI had only minor differences. The maximum CI was reached in about 2 days followed by a decrease until the end of the 10-day period. Throughout the whole period, the CI vs. time plots were considerably overlapping.

**Figure 5:**
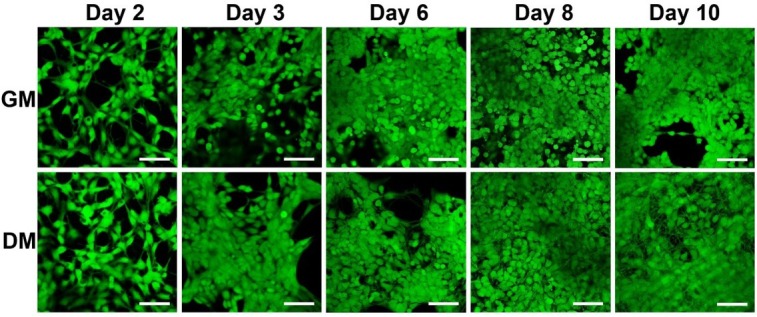
Fluorescence microscopy images of live stained (Calcein AM) hVM1 Bcl-X_L_ cells in growth medium (GM) and differentiation medium (DM). Initial cell density 120,000 cells/cm^2^. Time in days after cell seeding. (Scale bars: 50 μm)

In parallel with impedance monitoring, hVM1 Bcl-X_L_ cells were stained with calcein AM in order to evaluate the viability and morphological changes of the cells during the measurements (Figure 5). Live imaging confirmed that both proliferating and differentiating hVM1 Bcl-X_L_ cells were unaffected by the measurements.

When evaluating the morphological changes during the 10-day period, at first glance, both proliferating and differentiating cells appeared to behave similarly. Under both conditions, the cell density was clearly increasing, and toward the end of the period the cells were growing on top of each other. Previous experience of the behavior of hVM1 Bcl-X_L_ cells has indicated that proliferation still continues after replacement of the growth medium by differentiation medium, and if the cell population, proliferating or differentiating, becomes overconfluent, the cells start to lose their adherence on the growth substrate. At a closer look, toward the end of the 10-day period, the population of differentiating cells was, however, characterized by formation of a dense network of neural projections. Hence, the observed decrease in the CI after day 2, albeit apparently similar as indicated by the partially overlapping graphs, must in the case of differentiating cells have been caused by a combination of cell detachment and increased formation of neural projections. On the other hand, in the case of proliferating cells the cause was predominantly cell detachment due to overcrowding.

### Equivalent circuit analysis for discrimination of proliferating and differentiating neural stem cells

The results presented in the above section demonstrated that the calculated CI values allowed for distinction between proliferating and differentiating LUHMES cells first about 48 h after cell seeding (about 24 h after induction of neural differentiation). However, based on CI, the status of hVM1 Bcl-X_L_ cells could not be evaluated. As has been shown in previous applications related to stem cell differentiation, equivalent circuit analysis could provide more detailed information regarding the cellular status [9,10,21,22]. The data that was used above for CI-based analysis, was further evaluated to find a suitable equivalent circuit model capable of describing the behavior of the two different neural stem cell lines under proliferation and differentiation conditions. The goal of the performed equivalent circuit analysis was to provide an early distinction between proliferating and differentiating cells.

#### Choice of equivalent circuit model

For the study of epithelial and endothelial cells, Wegener *et al*. presented the first demonstration of equivalent circuit-based analysis [44]. Their equivalent circuit model comprised a capacitor and resistor in series, describing the impedance contribution of the electrode-electrolyte interface and the resistance of the conductive culture medium, respectively. The entire model had an additional parallel circuit composed of a resistor and capacitor that were assigned to describe the transepithelial/transendothelial resistance and the capacitance of the cell layer, respectively. The characteristic feature of the abovementioned study was a confluent monolayer of cells that strongly adhered to the electrode surface and formed strong cell-cell contacts.

In later studies, the parallel circuit of a resistor (R_cell_) and capacitor (C_cell_), originally presented by Wegener *et al*. as a general description of the cellular impedance contribution [44], has also been applied to analysis of impedance data recorded during stem cell differentiation [21,22]. R_cell_ has been assigned for the combined resistance of the cell membranes and intercellular contacts between adjacent cells. In order to account for varying adherence of the studied cells on an electrode surface, which depends on the cellular properties and the introduced adhesion factors (e.g. extracellular matrix) if needed, an additional resistor has been assigned. In different studies, the additional resistor has been placed either in parallel (denoted by R_seal_ [45, 46, 47]) or in series (denoted by R_extra_ [10]) with the circuit comprising R_cell_ and C_cell_. R_cell_ and R_extra_ are analogous to the differential equation derived parameters R_b_ and α^2^, respectively, originally introduced by Giaever and coworkers [15].

When characterizing the behavior of neural stem cells that are subjected to conditions that either promote proliferation or induce differentiation, the initial cell density is far below that of a confluent monolayer, as described above. The same is also valid when formation of neural projections becomes pronounced. The consequence is that at an early stage the cell layer does not fully cover the electrodes, which could make assignment of an equivalent circuit model more difficult than in studies that are based on a confluent monolayer of cells [19,44]. In order to find a suitable equivalent circuit model that could describe the cellular behavior, we used data recorded from IDEs that had a layer of proliferating stem cells that was as confluent as possible. Preliminary tests using an equivalent circuit comprising a resistor in series with a constant phase element (CPE) combined with a parallel circuit of a capacitor and resistor, indicated that the data could be fit. However, when using that equivalent circuit, analysis of spectra acquired during an entire proliferation and differentiation experiment indicated that it was not fully possible to distinguish between proliferating and differentiating hVM1 Bcl-X_L_ cells. In the case of LUHMES cells, the distinction was possible to the same extent as shown above for CI-based data presentation.

**Figure 6:**
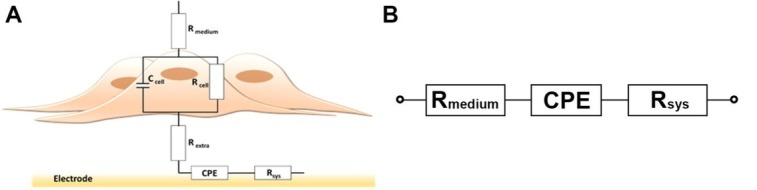
Equivalent circuit models for analysis of impedance spectra acquired **A**) in the presence and **B**) in the absence of cells. (*Cell specific parameters***: R_extra_**, **R_cell_**, **C_cell_**). For detailed description of the components, see the text.

Our preliminary data analysis, using an equivalent circuit model comprising R_extra_ in series with the parallel combination of R_cell_ and C_cell_ (Figure 6A), indicated, though, that for both stem cell lines, disregarding whether the impedance spectra had been acquired during an early or late stage during proliferation/differentiation, the behavior of the cells could be described. A CPE was assigned for the electrode-electrolyte interface impedance and the bulk medium conductivity was described by R_medium_. The additional series resistor, R_sys_, accounted for the characteristic resistance of the measurement system, including the possible contributions of interfacing to the miniaturized impedance analyzer.

**Figure 7:**
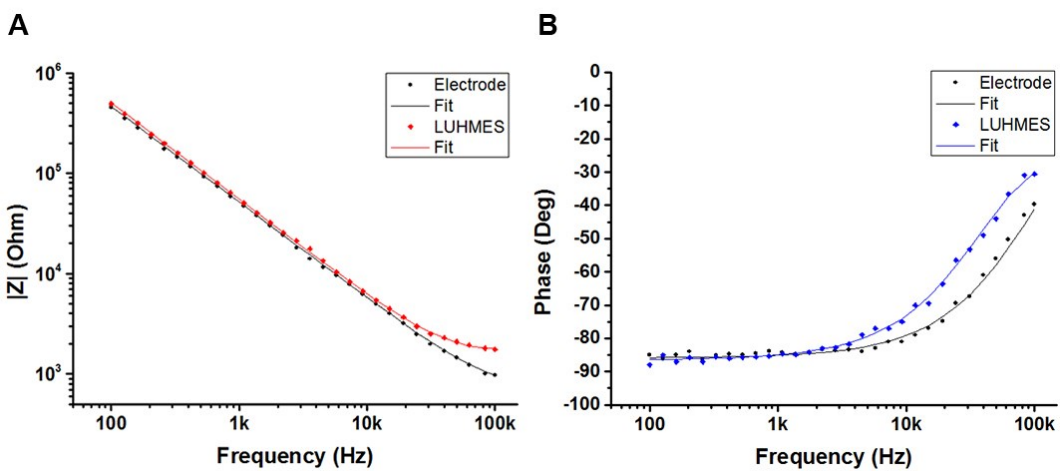
Example of typical Bode plots for an electrode and the same electrode 48 h after seeding of 60,000 LUHMES cells/cm^2^: **A**) impedance magnitude and **B**) phase angle. Solid lines show the nonlinear least squares fit of the experimental data to the equivalent circuit models of Fig. 6.

To determine the cell specific equivalent circuit parameters (R_extra_, R_cell,_ C_cell_), impedance spectra acquired in the absence of cells were first analysed using a simplified model (Figure 6B). For each electrode, the determined Z_CPE_, R_medium_ and R_sys_ were kept constant during the subsequent analysis of spectra that were acquired in the presence of cells. Figure 7 shows typical impedance spectra (presented as Bode plots for (A) impedance magnitude and (B) phase angle) acquired in the absence (electrode) and presence of LUHMES cells together with the resulting curve fitting. Impedance spectra were acquired in the range from 100 Hz to 100 kHz.

The number of decades in the spectra is limited and can influence the accuracy of the determined equivalent circuit parameters. As mentioned above, impedance spectra recorded using IDEs comprise the contribution of the interface impedance of both sides. The same applies to the influence of adhering cells. The equivalent circuit model described above is a generalization where the parameters have the contribution of cells on both sides of an IDE. Based on the applied frequency range and simplified equivalent circuit model, only comprising a collective impedance contribution instead of separating both sides of an IDE, the resulting equivalent circuit analysis serves as a tool for distinction between different cellular states.

### Characterization of cellular status

#### 1 LUHMES cells

**Figure 8:**
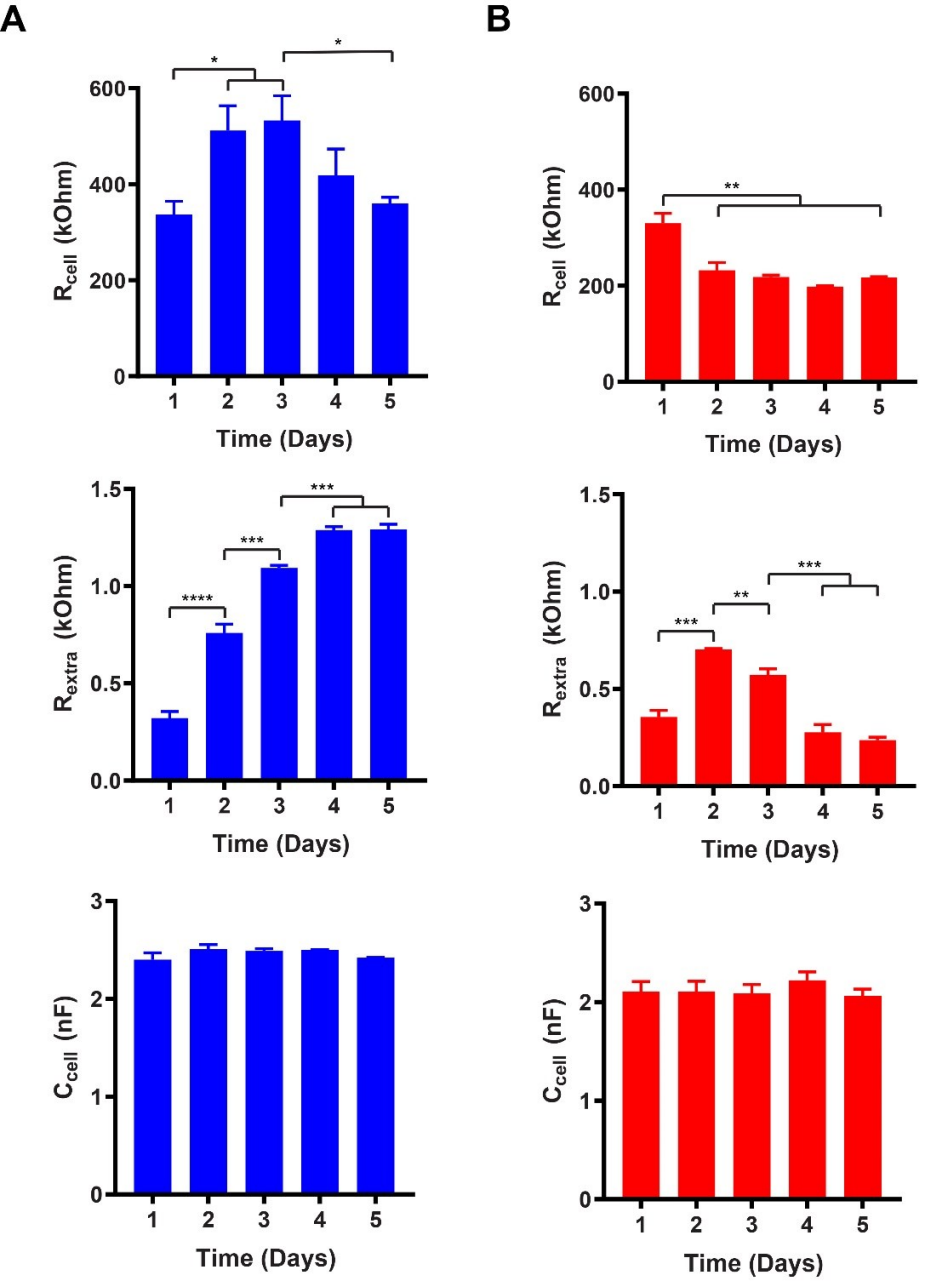
Summary of the cell specific equivalent circuit components (R_cell_, R_extra_, C_cell_) for **A**) proliferating and **B**) differentiating LUHMES cells (seeding density: 60,000 cells/cm^2^). Time in days after cell seeding. (Error bars: s.e.m., n = 6).

Figure 8 presents a summary of the variation of the three cell specific parameters over time (5 days) for both proliferating (A) and differentiating (B) LUHMES cells. For each parameter, day 1 represents the first 24 h after cell seeding. In the case of differentiating cells, it also corresponds to the time of medium change to induce differentiation.

For proliferating LUHMES cells, R_cell_ showed an initial increase (from day 1 to day 3), reaching a maximum value at day 3. Subsequently, R_cell_ decreased continuously, reaching at day 5 a level that was only slightly above that of day 1. R_extra_ increased significantly until it levelled off after day 4. C_cell_, on the other hand, did not undergo any statistically significant changes during the culture period. The initial increase in R_cell_ (until day 3) can be explained by the increased cell coverage and consequent formation of cell-cell contacts between the adjacent cells. Analogously, the increasing R_extra_ reflects the increasing number of strongly adhering cells. On the other hand, from day 3 to day 5, the contribution of R_cell_ differs significantly from that of R_extra_. By closer inspection of microscopic images (Figure 3), the behavior of R_cell_ and R_extra_ may be explained. The increased number of proliferating cells led to formation of intercellular spaces (i.e. partial disruption of the initially formed tight cell-cell contacts), decreasing R_cell_, whereas the entire cell layer remained, nevertheless, strongly adherent on the electrodes, contributing to the continuously increasing R_extra_.

In terms of R_cell_ and C_cell_, the behavior of proliferating LUHMES cells was similar to what has been reported by Seidel *et al*. for pNSC2 cells on Matrigel coated electrodes [10]. In the case of R_extra_, on the other hand, during the entire 14-day differentiation period, only minor fluctuations were reportedfor pNSC2 cells, whereas for LUHMES cells an increasing trend followed by leveling off was observed. The observed differences between LUHMES cells (on Geltrex^®^ coating) in our study and pNSC2 cells (on Matrigel coating) cannot be explained by the used electrode coatings since both Geltrex^®^ and Matrigel are similar extra cellular matrix coatings.

Differentiating LUHMES cells showed an initial decrease in R_cell_, leveling off after day 2, i.e. one day after the induction of differentiation, and showed only minor fluctuations during the remaining differentiation period. R_extra_ reached a maximum at day 2 followed by a continuous decrease until day 5. The greatest decrease was observed between day 3 and day 4. C_cell_ showed the same behavior that was observed for proliferating cells; no statistically significant changes occurred during the 5-day period. Although the induction of differentiation (at day 1) slows down proliferation and initiates formation of neural projections, some degree of proliferation has been observed until day 2.

The observed decrease in R_cell_ until day 2 can be attributed to the combination of these changes: The cell coverage on the electrodes still slightly increased; however, the initiated formation of neural projections resulted in morphological changes that widened the intercellular spaces, resulting in the net decrease in R_cell_. The initial increase in R_extra_ until day 2 can plausibly be explained by the continued proliferation, which still increased the number of adherent cells. Since the initial formation of neural projections was slow, the observed net effect was an increase in R_extra_. The subsequent decrease in R_extra_ clearly correlates with the increased formation of the neural projections, which do not strongly adhere on the electrodes. Microscopic images (Figure 3) also show that the formation of neural projections significantly increased from day 3 to day 4, explaining the strong decrease in R_extra_. Furthermore, general observations on LUHMES cell cultures with advanced differentiation indicated that the entire cell layers became weakly adherent. This means that aside from an increased amount of neural projections, the decrease in R_extra_ could be additionally contributed to by loosely adherent cell bodies. Comparison between proliferating and differentiating LUHMES cells indicates that the behavior of R_extra_ has a similar profile as CI for the two conditions. However, in combination with the additional information provided by R_cell_, it is clear that equivalent circuit analysis provides more detailed information of the cellular behavior and allows faster discrimination between proliferating and differentiating cells.

The observed overall behavior of LUHMES cells in terms of R_cell_ and R_extra_ was comparable to what has been reported by Seidel *et al*. for pNSC2 cells [10]. In the study, the decrease in R_extra_ was interpreted as possible degradation of the Matrigel coating. However, the most pronounced difference between LUHMES and pNSC2 cells was found in the behavior of C_cell_, which in the case of LUHMES cells remained more or less constant throughout the entire differentiation period. For pNSC2 cells, the statistically significant increase in C_cell_ toward the end of the differentiation period was explained in combination with the observed decrease in R_cell_ as a consequence of the increased cell membrane to cell volume ratio due to the formation of neural projections. Differentiation of LUHMES cells has been shown to increase β-III-tubulin synthesis, contributing to formation of the characteristic cytoskeleton of the neural projections [24]. In our study, both microscope images of differentiating LUHMES cells (Figure 3) and decrease in R_extra_ correlate with increased formation of neural projections. However, the fact that C_cell_ did not show any significant changes cannot be explained based on the present study.

#### 2 hVM1 Bcl-X_L_ cells

**Figure 9:**
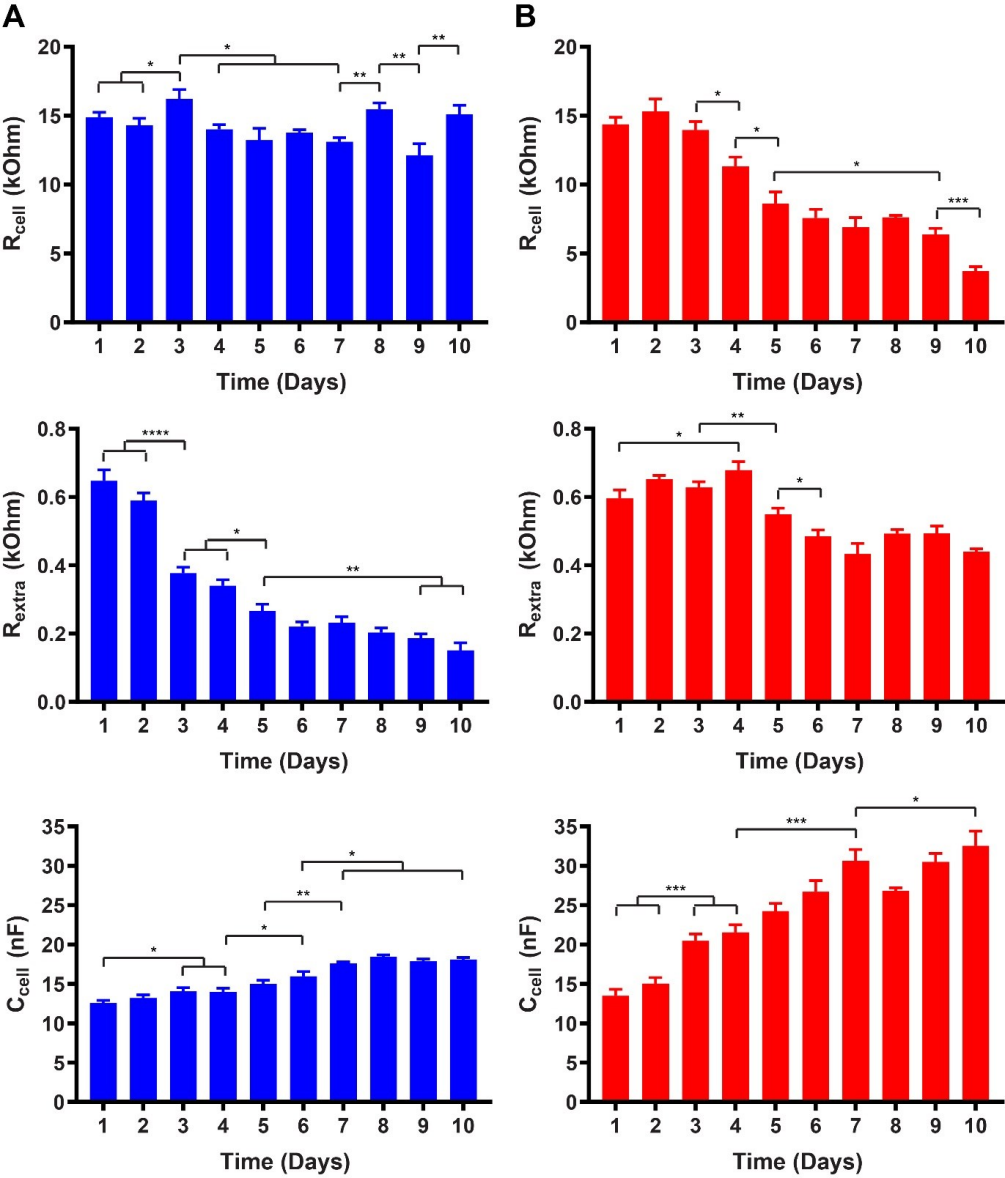
Summary of the cell specific equivalent circuit components (R_cell_, R_extra_, C_cell_) for **A**) proliferating and **B**) differentiating hVM1 Bcl-X_L_ cells (seeding density: 120,000 cells/cm^2^). Time in days after cell seeding. (Error bars: s.e.m., n = 6).

Figure 9 presents a summary of the variation of the three cell specific parameters over time (10 days) for proliferating (A) and differentiating (B) hVM1 Bcl-X_L_ cells. Day 1 represents the first 24 h after cell seeding, and in the case of differentiating cells, also corresponds to the time of medium change to induce differentiation.

For proliferating hVM1 Bcl-X_L_ cells, R_cell_ showed large fluctuations rather than any significant trend of increasing or decreasing values. On the other hand, R_extra_ decreased continuously until day 10 (the greatest decrease was observed between day 2 and day 3) while C_cell_ slightly increased until day 7, then leveling off. Microscopic imaging (Figure 5) during proliferation showed that the cell coverage on the electrodes continuously increased both laterally and vertically, i.e. while the area covered by the proliferating cells increased the cells also piled up on each other forming a multilayer. This led concomitantly to morphological changes manifested as more rounded and smaller cell bodies. The relatively constant, albeit fluctuating, R_cell_ can be understood as a contribution of multiple effects. The morphological changes caused by the increased lateral cell density decreased the tightness of the cell-cell contacts (increased gaps between the cells), leading to decreased R_cell_.

The voids formed between the cells were partially filled by the cells that grew on top of them. The combination of the two opposing effects may have plausibly resulted in the fluctuating R_cell_. Additionally, observations during cell proliferation indicated that the older the cultures became the more the entire cell layers were prone to detachment. This phenomenon may have contributed to an increased distance between the cells and the electrode surfaces, leading to the gradual decrease in R_extra_. The small, albeit statistically significant, increase in C_cell_ can be explained in combination with the gradually decreasing R_extra_ due to the observed tendency of the cell layers to detach.

During differentiation of hVM1 Bcl-X_L_ cells, R_cell_ remained relatively constant during the first 3 days followed by a gradual decrease until day 10. R_extra_ showed fluctuations until day 4 followed by a period of decrease, finally leveling off at day 6, albeit showing fluctuations. In the end of the 10-day period, the overall level of R_extra_ remained significantly higher than that of the proliferating cells. C_cell_ increased significantly until day 7, after which it started to show signs of leveling off. The overall level of C_cell_ increased to much higher values than for proliferating cells. hVM1 Bcl-X_L_ cells continued proliferation after day 1 when the medium was changed to induce differentiation [25,26]. This can also be seen in the microscopic images shown in Figure 5. This may explain the initial period of relatively constant value of R_cell_ as was seen in the case of proliferating cells. The following decrease in R_cell_ is clearly caused by the starting formation of neural projections that create voids between the cells. Although the cells still continued to proliferate a few days after induction of differentiation, the formed cell layer did not become as highly packed and was not equally prone to detachment as in the case of proliferating cells. On the contrary, even the neural projections seemed to adhere on the electrodes. These observations can directly explain the smaller decrease in R_extra_ in comparison with the proliferating cells. The behavior of R_cell_ and R_extra_, explained above, combined with the fact that the formation of neural projections increased the cell membrane to cell volume ratio can also explain the observed increase in C_cell_ similarly as has been reported for differentiating pNSC2 cells [10].

Comparison between proliferating and differentiating hVM1 Bcl-X_L_ cells indicates that all the three cell specific parameters have a different behavior. Hence, equivalent circuit analysis clearly allows effective and early discrimination between the two conditions unlike the CI-based analysis, which was essentially unable to distinguish between them.

## Conclusion

Using impedance-based monitoring, we present the first characterization of two human mesencephalic fetal neural stem lines (LUHMES and hVM1 Bcl-X_L_) that have been developed for the study of the pathogenesis of Parkinson’s disease and its treatment by cell replacement therapy (CRT). The widely adopted impedance magnitude analysis was able to provide a general distinction between proliferating and differentiating LUHMES cells, whereas in the case of hVM1 Bcl-X_L_ cells no distinction was possible. Hence, the presented results highlight the value of equivalent circuit-based analysis as an excellent tool to distinguish between proliferating and differentiating neural stem cells when impedance magnitude does not provide sufficient information. Such data analysis allows to elucidate the changes in the cellular behavior related to cell morphology, cell-cell contacts, and adhesion to the growth substrate (electrodes). Moreover, our findings demonstrate that impedance monitoring provides such information much earlier than microscopic imaging. This study demonstrates the importance of impedance assays in providing additional insight into the differentiation process of stem cells and forms a basis for the development of future screening assays. Such impedance-based assays in stem cell biology can exactly be aimed at development of stem cell lines for CRT, elucidation of protocols for epigenetic control of stem cells, and drug screening.

## Supplementary Material

### S-1 Normalized impedance as a function of frequency

The result indicates that the highest value was obtained for 100 kHz. Impedance at 100 kHz was then used to calculate the Cell Index in the subsequent experiments.

## S-2 Dependence of Cell Index on cell seeding density of hVM1 Bcl-XL cells

The results indicate that 120,000 cells/cm^2^ is the optimal cell seeding density for hVM1 Bcl-X_L_ cells. All subsequent experiments shown in the paper were performed using this cell seeding density.
